# Analysis of Substance Use Disorder Treatment Admissions in the US by Sex and Race and Ethnicity Before and During the COVID-19 Pandemic

**DOI:** 10.1001/jamanetworkopen.2022.32795

**Published:** 2022-09-22

**Authors:** Jonathan H. Cantor, Christopher M. Whaley, Bradley D. Stein, David Powell

**Affiliations:** 1 Department of Healthcare Delivery, RAND Corporation, Santa Monica, California; 2 Department of Healthcare Delivery, RAND Corporation, San Francisco, California; 3 Department of Healthcare Delivery, RAND Corporation, Pittsburgh, Pennsylvania

## Abstract

This cross-sectional study examines the changes in treatment admissions for substance use disorder during the COVID-19 pandemic by sex and race and ethnicity.

## Introduction

The COVID-19 pandemic has led to increases in the number of fatal drug overdoses^1^ and self-reported substance use disorder (SUD).^2^ Despite these increases, few studies have examined SUD treatment admissions during the pandemic, with studies focusing on state-specific estimates^3^ or inferring use through national mobility data.^4^ To more comprehensively examine the surge in drug overdose deaths, we quantified changes in national SUD treatment before (2017-2019) and during (2020) the COVID-19 pandemic.

## Methods

We used 2017-2020 data from the Treatment Episode Data Set,^5^ which is client-level data for SUD treatment admissions from state agency data systems as reported by facilities that receive state funds or block grants. We quantified the number of SUD treatment admissions for each state and the District of Columbia, by race and ethnicity (as categorized in the Treatment Episode Data Set), and by sex per 10 000 population aged 12 years and older. We acquired population data from the Surveillance, Epidemiology, and End Results database. We mapped 2019-2020 changes in the number of treatment admissions by state and calculated changes in the number of SUD treatment admissions by race and ethnicity and sex between 2017 and 2020. Using a cross-sectional design, we collected anonymized, publicly available data on the number of state-level treatment admissions, and the study was deemed exempt by the RAND Human Subjects Protection Committee. This study followed the Strengthening the Reporting of Observational Studies in Epidemiology (STROBE) reporting guideline. Analyses were conducted using Stata, version 17 (StataCorp LLC).

## Results

**Figure 1.**
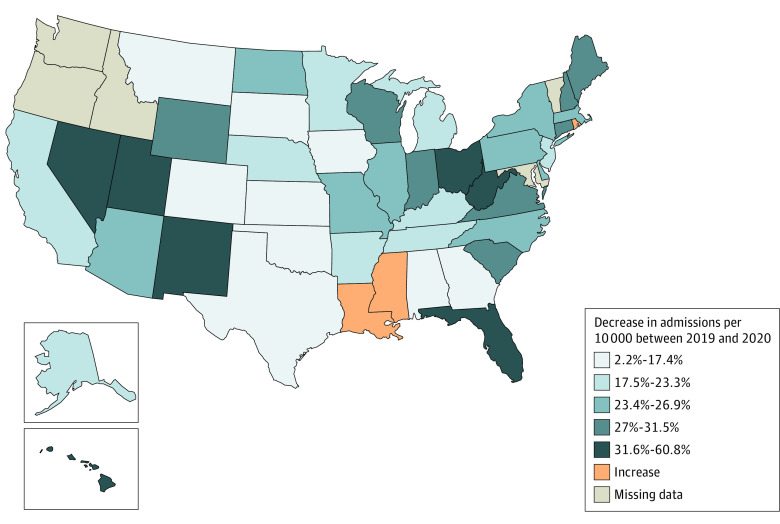
Percent Change in Number of Substance Use Disorder Treatment Admissions per 10 000 Between 2019 and 2020 The largest decreases were noted in New Mexico (60.8%), Hawaii (54.5%), Washington, DC (44.9%), Nevada (41.5%), and West Virginia. Admissions increased in Rhode Island (7.9%), Louisiana (3.7%), and Mississippi (0.8%).

The largest state-level per 10 000 population decreases in treatment admissions were in New Mexico (60.8%), Hawaii (54.5%), District of Columbia (44.9%), Nevada (41.5%), and West Virginia (33.3%) (Figure 1). In contrast, treatment admissions increased in Rhode Island (7.9%), Louisiana (3.7%), and Mississippi (0.8%).

**Figure 2.**
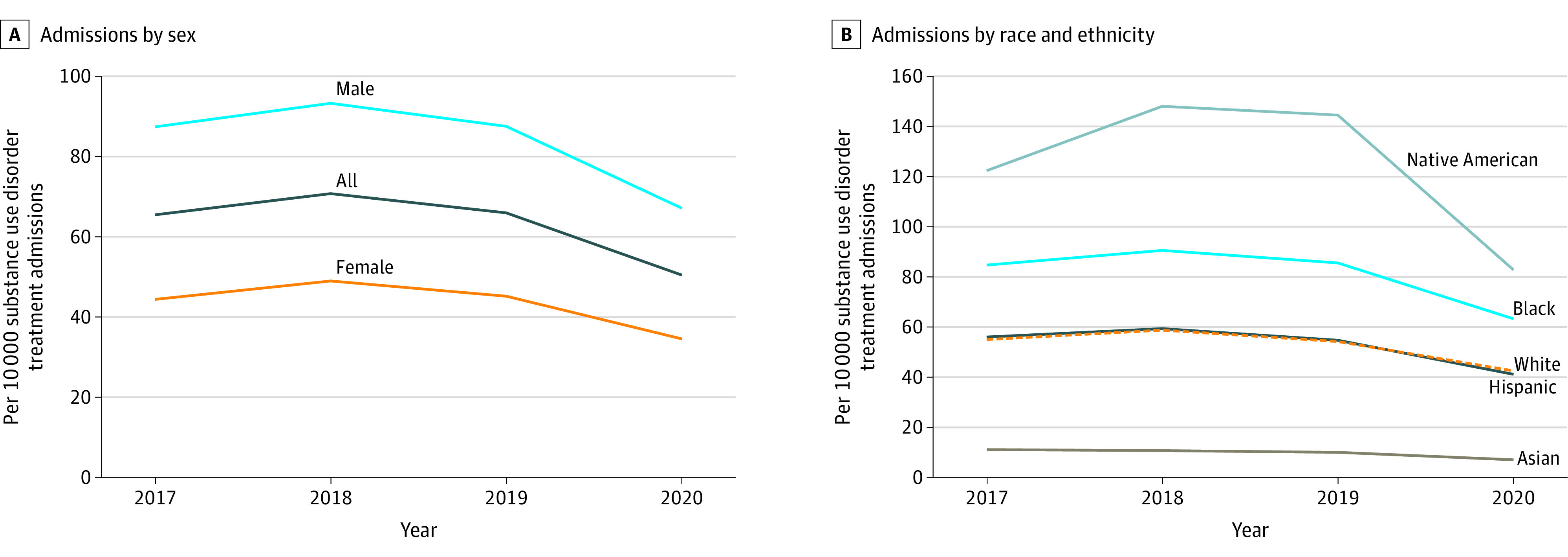
Trends in Treatment Admissions per 10 000 Population Between 2017 and 2020 Treatment admissions data by sex (A) and race and ethnicity (B) were obtained from the Treatment Episode Data Set between 2017 and 2020. The number of admissions was adjusted per 10 000 population from the Surveillance, Epidemiology, and End Results database.

Before 2020, the number of treatment admissions per 10 000 remained relatively stable. However, in 2020, the number of treatment admissions decreased from 65.9 per 10 000 in 2019 to 50.4 per 10 000 in 2020, a relative reduction of 23.5% (Figure 2A). The decrease was larger for men (87.5 to 67.1 per 10 000) compared with women (45.1 to 34.5 per 10 000). All racial and ethnic groups experienced a decrease in treatment admissions, with the largest decrease observed for Native American individuals (144.5 to 82.8 per 10 000) followed by Black (85.5 to 63.3 per 10 000), Hispanic (54.7 to 41.1 per 10 000), White (54.2 to 42.5 per 10 000), and Asian (10.0 to 7.1 per 10 000) individuals (Figure 2B).

## Discussion

We noted a 23.5% national decrease in treatment admissions and decreases in almost all states. These decreases are especially noteworthy given evidence of increases in SUD and overdose death rates during the same period. The largest decreases were found for Native American individuals. We identified larger decreases for Black individuals compared with White and Asian individuals. Native American individuals experienced the largest increases in drug overdose deaths during the pandemic, and Black individuals had higher overdose deaths than White individuals.^1^

This study has limitations. First, it did not include Idaho, Oregon, Maryland, Vermont, and Washington. Second, we were not able to examine possible mechanisms for the differences in the size of the decreases. Future work is needed to examine possible reasons for the differences, including policy (eg, elective procedure bans and shelter-in-place policies) changes. Third, we did not examine the types of treatment provided.^6^ To our knowledge, this is the first national study to report decreases in the number of SUD treatment admissions during the COVID-19 pandemic and provides a possible reason for the increase in drug overdose deaths.
